# Malocclusion, dental aesthetic self-perception and quality of life in a 18 to 21 year-old population: a cross section study

**DOI:** 10.1186/1472-6831-13-3

**Published:** 2013-01-07

**Authors:** Dikson Claudino, Jefferson Traebert

**Affiliations:** 1 Post-Graduation Programme on Health Sciences, Universidade do Sul de Santa Catarina, Tubarão, SC, 88704-900, Brazil

**Keywords:** Malocclusion, Oral health, Aesthetics, Quality of life

## Abstract

**Background:**

Aesthetic alterations in the face can be self-perceived and can affect quality of life. For young people, physical attractiveness is an important factor affecting social relationships. The aim of this study was to estimate the prevalence of malocclusion, identify the most common types and test its association with oral aesthetic self-perception in 18 to 21 year-old population of male young adults.

**Methods:**

A cross-sectional study was carried out involving 138 Brazilian Army soldiers. Data collection included socio demographic profile, malocclusion status through the Dental Aesthetic Index (DAI) and oral aesthetic self-perception as indicated by the Oral Aesthetic Subjective Impact Scale (OASIS). The chi-square and Fisher’s exact test were used to test for homogeneity of proportions. The stepwise multivariate logistic regression analysis was used to test for the relationship between the poorer oral aesthetic self-perception and parental and soldier’s education, *per capita* income, history of caries in all teeth and only on anterior teeth, dental trauma, previous orthodontic treatment and malocclusion.

**Results:**

The prevalence of malocclusion was 45.6%. Incisor teeth crowding and misalignment of lower incisors were the most common types of malocclusions. A statistically significant and independent association between malocclusion and poorer oral aesthetic self-perception in the multivariate analysis was observed. Subjects with severe malocclusion conditions showed 88% higher prevalence [prevalence ratio =1.88 (95% CI, 1.30 – 2.72); p = 0.001] of poorer aesthetic self-perception comparing to those with minor malocclusion.

**Conclusions:**

A high prevalence of malocclusion was observed. The young adults presenting severe malocclusion had a higher and independent prevalence of poorer oral aesthetic self-perception.

## Background

Despite the lack of consistent evidence, that malocclusion can affect psychosocial wellbeing in the long term [1] it has been claimed the facial features, especially oral aesthetics had a potential to influence self-perceived appearance [2] especially during the phase of life when there is intense social and affective interaction.

For young people physical attractiveness is an important factor affecting social relationships [3]. Thus, aesthetic alterations in the face can be self-perceived and can affect quality of life [4-6]. For instance, among young adults in Finland, the primary motives for orthodontic treatment were to improve dental appearance and attitudes toward malocclusion [7]. In a Brazilian study involving adolescents, youths who had completed orthodontic treatment reported fewer oral health impacts related in smiling, laughing and showing teeth without embarrassment [8].

Reduced susceptibility to dental caries and trauma, periodontal disease and temporomandibular disorders have been mentioned as possible benefits of orthodontic treatment, but research has consistently failed to provide solid evidence of social or psychological benefits from orthodontic treatment [9].

Therefore, the objective of this study was to estimate the prevalence of malocclusion, identify the most common types of malocclusions and test the association between malocclusion and oral aesthetic self-perception in a sample of 18 to 21 year-old male population.

## Methods

A cross-sectional study was carried out involving a sample of Brazilian Army soldiers aged between 18 and 21 years in the city of Tubarão, in the Southern Brazilian State of Santa Catarina. The research project was submitted to and approved by the Research Ethics Committee of the *Universidade do Sul de Santa Catarina*, protocol 09.616.4.02.III, and those who agreed to participate signed a consent form.

### Study sample

Overall, 150 soldiers of the Brazilian Army unit of the city were invited to participate in the present study. Of the total of 150 subjects, 138 were examined and interviewed, resulting in a response rate of 92.0%. Mean age was 19.4 years (SD = 1.36). Data collection was carried out at a dental office. The subjects responded to a structured interview regarding socio demographic characteristics such as family income, education level and previous orthodontic treatment.

#### Oral clinical examination

After the interview, clinical data were collected through oral examination. The examiner used wooden spatulas, clinical mirrors and periodontal probes previously sterilized. For examination, individuals remained reclined in the dental chair under both artificial light from the equipment and natural light from the window.

#### Assessment of dental aesthetic self-perception

Data on oral aesthetic self-perception was collected through the Oral Aesthetic Subjective Impact Scale (OASIS). This indicator was developed by Mandall et al. [10] and validated in Brazil by Pimenta and Traebert [11]. It consists of five questions regarding concerns on self-perceived oral appearance to be answered in a seven-point Likert-type rating scale.

#### Malocclusion severity assessment

Data on malocclusion was collected through the Dental Aesthetic Index (DAI), according to WHO [12] criteria. DAI assessment includes ten parameters of dentofacial structure relating to tooth positioning and the relationship between maxillary and mandibular arches. It classifies dental malocclusion as mild (or normal occlusion); definite; severe or very severe conditions [13].

#### Possible confounder variables

To control for possible confounding clinical variables, data on dental trauma [14] and dental caries (history of caries measured by the DMF-T index: number of decayed, missing due to caries and filled teeth) [12] were also collected.

### Examiner calibration

To ensure the diagnostic reliability, calibration of the clinical examiner was performed before data collection with a double-oral examination of ten individuals in the sample, at five-day intervals. The kappa statistic was used as a measure of reliability with minimum allowed kappa equal to 0.7 for each clinical situation studied. In addition, during clinical examinations, 10% randomly selected individuals in the sample underwent double examination to ensure the reliability of diagnostic during data collection.

### Statistical analysis

The Chi-square (χ^2^) and Fisher’s exact test were used to test for the relationship between the poorer oral aesthetic self-perception and parental and soldier’s education, *per capita* income, history of caries in all teeth and only on anterior teeth, dental trauma, previous orthodontic treatment and malocclusion. The significance level was set at p < 0.05. These tests were chosen because it was decided to dichotomize the variables in order to carry out the logistic regression analysis. The stepwise multivariate logistic regression analysis [15] was used to adjust the association between the poorer oral aesthetic self-perception and parental and soldier’s education, *per capita* income, DMF-T in all teeth and only on anterior teeth, dental trauma, previous orthodontic treatment and malocclusion. Statistically significant variables with a p-value <0.20 in the bivariate analysis were entered into the logistic model, starting with the variable that had the highest level of statistical significance and following in descending order. Odds ratios (OR) and confidence intervals (95%) were converted into prevalence ratios (PR) as recommended by Schiaffino et al. [16]. The data were analyzed using SPSS 16.0 (SPSS, Inc., Chicago, IL, USA)

The dependent variable was the poorer oral aesthetic self-perception as assessed by the OASIS with the cutoff point at the 75th percentile. Scores between 17 and 29 accounted for poorer self-perception. The independent variables were parental and soldier’s education (cutoff point at eight years of schooling, since it represents the final of the primary degree of formal education in Brazil); *per capita* income (cutoff point at the distribution’s median – BR *reais* of 525.00); history of caries in all teeth and only on anterior teeth (cutoff DMF-T zero or non-zero); dental trauma (present or absent); previous orthodontic treatment (yes or no); and malocclusion assessed by the DAI (cutoff point - DAI scores ≤ 30: minor malocclusion, scores > 30: severe malocclusion).

## Results

Regarding education, 89.8% of the surveyed subjects, and 46.4% of their fathers and 49.1% of their mothers had more than eight years of schooling. Data on family income showed that the median *per capita* was BR *reais* of 525.00 (about U$ 316.00 - September 2011).

The results in this study showed that 45.6% (95% CI, 37.3-53.9) of the surveyed subjects had definite, severe or very severe malocclusion. There was a high percentage of dental crowding in one or both dental arches (55.0%). Misalignment of teeth in the mandibular arch was 47.8%. These and other conditions relating to malocclusion are presented in Table 1.

**Table 1:** Distribution and percentage of malocclusion according to DAI

**MALOCCLUSION**	**n**	**%**
**SEVERITY (DAI)**		
Normal occlusion or light malocclusion^1^	75	54.4
Definite malocclusion^2^	26	18.8
Severe malocclusion^3^	24	17.4
Very severe malocclusion^4^	13	9.4
**COMPONENTS (DAI)**		
**Missing teeth**		
No missing tooth	134	97.1
One missing tooth	4	2.9
**Crowding**		
Without crowding	62	45.0
Crowding in one arch only	46	33.3
Crowding in both arches	30	21.7
**Incisal spaces**		
No spaces	113	81.9
Spaces in one arch only	22	15.9
Spaces in both arches	3	2.2
**Incisal diastema**		
No diastema	120	87.0
1 to 3 mm	18	13.0
**Maxillary misalignment**		
No misalignment	106	76.9
1 to 3 mm	30	21.7
> 3 mm	2	1.4
**Mandibular misalignment**		
No misalignment	72	52.2
1 to 3 mm	66	47.8
***Overjet***		
0 – 3 mm	111	80.5
4 – 6 mm	27	19.5
**Anterior open bite**		
No open bite	128	92.9
1 to 5 mm	10	7.1
**Molar relationship**		
Normal	82	59.4
Half-cusp deviation	20	14.5
Full-cusp deviation	36	26.1
**TOTAL**	**138**	**100.0**

^1^DAI scores ≤ 25, ^2^DAI score of 26–30, ^3^DAI score of 31–35, ^4^DAI score ≥36.

Clinical data also showed that 24.6% (95% CI, 17.4-31.8) of the subjects had a DMF-T score equal to zero; the mean DMF-T was 4.12 (SD = 4.54). Dental trauma showed a prevalence of 29.0% (95% CI, 21.4-36.6). Of the 138 surveyed subjects, 25.4% (95% CI, 18.1-32.7) received previous orthodontic treatment.

Respondents showed great concern regarding oral aesthetic. In response to the question “*How do you feel about the appearance of your teeth*?” the median score was 6 in a seven-point scale. On the other hand, with reference to social constraint, assessed by the question “*Do you try to avoid smiling because of the appearance of your teeth*?” the median score was 1; 79.7% scored the lowest possible value of the scale (1) while only 4.3% scored the highest value (7) (Table 2). The mean OASIS score showed to be 14.3 (SD = 4.6); the median was 14.

**Table 2:** Distributions of OASIS components

QUESTIONS/SCORES*	**n**	**%**
**How do you feel about the appearance of your teeth?**		
1	2	1.4
2	2	1.4
3	0	0.0
4	15	11.0
5	42	30.4
6	42	30.4
7	35	25.4
**Have you found that other people have commented on the appearance of your teeth?**		
1	20	14.5
2	19	13.8
3	14	10.1
4	12	8.7
5	39	28.3
6	20	14.5
7	14	10.1
**Have you found that other people have teased you about the appearance of your teeth?**		
1	99	71.8
2	17	12.3
3	6	4.3
4	6	4.3
5	4	2.9
6	3	2.2
7	3	2.2
**Do you try to avoid smiling because of the appearance of your teeth?**		
1	110	79.8
2	9	6.5
3	3	2.2
4	3	2.2
5	6	4.3
6	1	0.7
7	6	4.3
**Do you ever cover your mouth because of the appearance of your teeth?**		
1	124	90.0
2	2	1.4
3	4	2.9
4	5	3.6
5	2	1.4
6	0	0.0
7	1	0.7
**TOTAL**	**138**	**100.0**

*Scores 1 to 7 represent a seven-point Likert scale, where the score 1 indicates the best perception of dental appearance and score 7 indicates the poorest.

Table 3 shows the findings for an association between oral aesthetic self-perception and socio demographic and oral clinical variables. It can be observed that only the DAI showed a statistically significant association (p < 0.001).

**Table 3:** Association between OASIS and socio-demographic and oral clinical data

**VARIABLE**	**POORER SELF-PERCEPTION**^**1**^**n (%)**	**BETTER SELF–PARCEPTION**^**1**^**n (%)**	**χ**^**2**^	**p**
***Per capita *income**				
**(median - BRL)**				
≤525.00	19 (27.5)	50 (72.5)	0.996	0.318
>525.00	14 (20.3)	55 (79.7)		
**Soldier’s education**				
**(years of schooling completed)**				
≤8 years	4 (28.6)	10 (71.4)	0.186	0.666
>8 years	29 (23.4)	95 (76.6)		
**Mother’s soldier education**				
**(years of schooling completed)**				
≤8 years	17 (25.8)	49 (74.2)	0.090	0.765
>8 years	16 (23.5)	52 (76.5)		
**Father’s soldier education**				
**(years of schooling completed)**				
≤8 years	14 (24.1)	44 (75.9)	0.012	0.912
>8 years	16 (25.0)	48 (75.0)		
**DMF-T**				
≠ zero	25 (24.0)	79 (76.0)	0.004	0.952
Zero	8 (23.5)	26 (76.5)		
**DMF-T in anterior teeth**				
≠ zero	6 (28.6)	15 (71.4)	0.295	0.587
Zero	27 (23.1)	90 (76.9)		
**DAI**				
Severe malocclusion^2^	17 (45.9)	20 (54.1)	13.488	<0.001
Minor malocclusion^3^	16 (15.8)	85 (84.2)		
**Previous orthodontic treatment**				
Yes	10 (28.6)	25 (71.4)	0.559	0.455
No	23 (22.3)	80 (77.7)		
**Dental trauma**				
Present	12 (30.0)	28 (70.0)	1.014	0.316
Absent	21 (21.9)	75 (78.1)		

^1^Cutoff point: 75^th^ percentile - scores between 17 and 29 - poorer self-perception; scores between 5 and 16 - better self-perception. ^2^DAI scores > 30. ^3^DAI scores ≤ 30.

Results of logistic regression showed that DAI remained statistically significant (p = 0.001) after adjusted for *per capita* income, DMF-T in anterior teeth and dental trauma. Individuals with severe malocclusion showed 88.0% higher prevalence [prevalence ratio =1.88 (95% CI, 1.30 – 2.72); p = 0.001] of poor aesthetic self-perception compared to those who had minor malocclusion status (Table 4).

**Table 4:** **Logistic regression results indicating associations between poorer OASIS and *per capita *income and oral clinical conditions**

**VARIABLES**	**PR**^**a**^**(95% CI)**	**p**	**PR**^**b**^**(95% CI)**	**p**
***Per capita *income (median – BR *reais*)**		0.318		0.736
≤525.00	1.10 (0.91 – 1.33)		1.09 (0.66 – 1.80)	
>525.00	1.00		1.00	
**DMF-T in anterior teeth**		0.587		0.617
≠ zero	1.08 (0.82 – 1.43)		1.18 (0.62 – 2.25)	
Zero	1.00		1.00	
**DAI**		<0.001		0.001
Severe malocclusion^1^	1.56 (1.22 – 2.00)		1.88 (1.30 – 2.72)	
Minor malocclusion^2^	1.00		1.00	
**Dental trauma**		0.316		0.310
Present	1.12 (0.90 – 1.40)		1.29 (0.79 – 2.11)	
Absent	1.00		1.00	

^a^Bivariate analysis; ^b^Adjusted analysis.

^1^DAI scores > 30; ^2^DAI scores ≤ 30.

Hosmer & Lemeshow test (p = 0.338).

## Discussion

This study found a high prevalence of malocclusions classified as severe or very severe (26.8%) which corresponds to DAI scores equal to or greater than 31. According to Cons et al. [13] these scores indicate the need for a highly desirable or mandatory orthodontic treatment. This prevalence rate is close to that found by Hamanci et al. [17] in a study that investigated the relationship between the severity of malocclusion, self-perception of satisfaction with oral appearance among young adults. These authors found that 21.5% of the surveyed subjects had severe or very severe malocclusion, statistically associated with dissatisfaction with oral appearance.

The results of this study showed no statistically significant association between oral aesthetic self-perception and previous orthodontic treatment. These data are in agreement with those found by Mandall et al. [10] According to the authors, while individuals with minor normative malocclusion can have a poorer oral aesthetic self-perception, orthodontically treated individuals do not show a statistically significant difference in relation to oral aesthetic self-perception when compared with untreated individuals. Kenealy et al. [18] in a 20 year follow-up study concluded that there was little evidence to support that orthodontics improves long-tem psychological health. Also, Shaw et al. [19] in a cohort study pointed out that orthodontic treatment when needed did not lead to psychological difficulties in later age. Arrow et al. [20] found no statistically significant association between occlusal status at adolescence and quality of life at adulthood. They concluded the receipt of fixed orthodontic treatment was not associated with oral health-related quality of life, but appeared to be negatively associated with self-esteem and satisfaction with life.

Logistic regression analysis demonstrated a statistically association between malocclusion and oral aesthetic self-perception. Individuals with severe malocclusion had a poorer aesthetic self-perception when compared to individuals with minor malocclusion rates. These results are consistent with findings reported by other authors [21-23] who found, among other things, that individuals with worse dental occlusion conditions, as measured by clinical normative indicators, had a poorer oral aesthetic self-perception. This can be hypothetically explained by the fact that individuals can more easily identify severe malocclusion conditions. While minor malocclusions do not cause negative perception of dental aesthetics, severe malocclusions have the potential to be more easily recognized by individuals as harmful to oral aesthetics.

Anterior incisor abnormalities can affect the individuals’ oral aesthetics. Facial and dental attractiveness represents an important element of quality of life [4-6]. Due to easy viewing in comparison to the back teeth, negative aesthetic alterations in anterior teeth easily lead to dissatisfaction with oral aesthetics. Aesthetic alterations relating to the incisor positioning are strongly related to the demand for orthodontic treatment in adults [24] to achieve a better oral aesthetic. In a literature review, Zhang and McGrath [25] concluded that malocclusion and its treatment could affect psychological health in terms of self-concept. According to a review [6] it was found that patients focus on esthetics and social aspects of oral health-related quality of live as reasons for seeking orthodontic treatment. However, undergoing orthodontic intervention has been found to enhance some aspects of quality of life, particularly esthetics, but not necessarily social acceptance. Moreover, self-esteem does not appear to be affected in long term.

Individuals who perceive themselves as having a great need for orthodontic treatment are those who had a poor self-perception of oral aesthetics and low self-esteem [21]. In a cross-sectional study carried out with adolescents it was found that individuals who had the worst malocclusion conditions also had the poorest oral aesthetic self-perception, with aesthetic impact expressed by the constraint on smiling or showing teeth [26]. In two surveys conducted with a similar population of the present study, malocclusions affecting the anterior dental arches, such as crowding of incisors and severe overjet, were found to be associated with the self-report of impacts on the quality of life [3,27].

In interpreting the outcome of this study, it is important to bear in mind the limitations of the present study. Its cross-sectional design prevents establishing any causal relationship between malocclusion and the poor self-perception of oral aesthetics, making it impossible to determine whether the associations found preceded or followed the occurrence of the outcome. As the controversy about the impact of malocclusion and its treatment remains [25] more studies with longitudinal designs are necessary with rigorous assessment of malocclusion and its impact on quality of life. The weakness of aesthetic orthodontic indices and the subjectivity, which is associated with their use, have been mentioned previously [1]. The author also points out that DAI does not represent all occlusal traits.

Another limitation is the possible homogenized sample of this study. The selection of young males for engagement in the mandatory military service includes a rigorous health evaluation, including assessment of oral health. This process excludes those with poor oral health conditions, thus resulting in a homogenous sample. In addition, the inclusion in the sample of only males aged between 18 and 21 years limits extrapolation of results to other populations; therefore findings cannot be applied to the general population.

## Conclusions

A high prevalence of malocclusion was observed. The young adults presenting severe malocclusion had a higher and independent prevalence of poorer oral aesthetic self-perception.

## Competing interests

The authors declare that they have no competing interests.

## Authors’ contributions

The two authors contributed to the conduct of the study in its all phases. Both authors read and approved the final version of the manuscript.

## Pre-publication history

The pre-publication history for this paper can be accessed here:

http://www.biomedcentral.com/1472-6831/13/3/prepub
